# Search mode, not the attentional window, determines the magnitude of attentional capture

**DOI:** 10.3758/s13414-022-02582-4

**Published:** 2022-10-07

**Authors:** Dirk Kerzel, Stanislas Huynh Cong

**Affiliations:** Faculté de Psychologie et des Sciences de l’Education, Université de Genève, 40 Boulevard du Pont d’Arve, 1205 Geneva, Switzerland

**Keywords:** Visual search, Attentional capture, Search mode, Singleton search, Feature search

## Abstract

A salient color distractor is known to capture attention during search for a less salient shape target, but the mechanisms underlying attentional capture are debated. Theeuwes (2004, *Psychonomic Bulletin & Review, 11*(1), 65–70) argued that attentional capture depends on the size of the attentional window. If the attentional window is large, search is efficient and attentional capture should be stronger because the distractor is more likely to be inside the window. Consistently, we found higher search efficiency and more attentional capture in singleton than in feature search. However, differences in attentional capture only occurred when singleton and feature search were performed by different groups of participants, but not when singleton and feature search occurred unpredictably in the same group of participants. This result contradicts the attentional window account because search efficiency was always higher in singleton than in feature search. Rather, the results support search mode theory, which claims that participants looked for the most salient stimulus in singleton search (“singleton detection mode”), which resulted in more capture by the salient color distractor. When search types varied unpredictably, it was impossible to apply a consistent search strategy, which eliminated the differences between singleton and feature search. Further, we manipulated search efficiency by target–nontarget similarity. With dissimilar nontargets, the target was salient and search efficiency was high. Therefore, the attentional window account predicts more capture. However, we found the opposite result in singleton search and no difference in feature search. Taken together, these observations are inconsistent with the attentional window account but support search mode theory.

## Introduction

We must select goal-relevant stimuli from the overwhelming amount of information arriving at our sensory organs. How selection is accomplished has been a matter of debate for a long time (Awh et al., 2012; Kruger et al., 2017; Lamy et al., 2012; Luck et al., 2021). The contingent capture paradigm developed by Folk et al. (1992) has provided evidence that attentional selection is controlled by top-down factors, such as search goals (reviewed by Burnham, 2007; Busel et al., 2020). In contrast, the additional singleton paradigm developed by Theeuwes (1991, 1992) provided evidence for the implication of bottom-up factors, such as stimulus saliency. In the most common variant of the additional singleton paradigm, observers search for a shape singleton. The shape singleton is salient because it is unique from the nontargets. On some trials, one of the nontarget shapes is shown in a singleton color. The color singleton acts as a distractor because it is more salient than the shape singleton. Reaction times (RTs) on distractor-present trials were found to be longer than RTs on distractor-absent trials, suggesting that the color distractor captured attention because of its larger saliency (reviewed by Theeuwes, 2010). As explained below, this idea was later qualified by Bacon and Egeth’s (1994) search mode theory and Theeuwes’ (2004) attentional window account. In the present contribution, we will test the attentional window account against the competing search mode theory through manipulations of search efficiency and observer expectations. We find that a central prediction of the attentional window account—namely, that attentional capture decreases if search becomes less efficient, does not hold.

### Search mode theory

In search mode theory, Bacon and Egeth (1994) argued that attentional capture depended on the type of search performed by the participant. In singleton search, participants search for a unique shape among equal nontargets (e.g., a diamond among circles). Even though it is in principle possible to search for the specific shape of the singleton, Bacon and Egeth pointed out that it is also possible to look for a salient shape. They referred to this search strategy as “singleton detection mode.” As a consequence of singleton detection mode, attention may be captured by other salient stimuli, such as a color distractor that is presented on some of the trials. However, attentional capture can be prevented by forcing participants to search for the exact target features. This “feature search mode” can be induced by adding unique nontarget shapes to the search display. With other unique shapes in the display, the target is no longer a singleton and can no longer be located by searching for a salient stimulus. In studies that were closely modeled on the feature search condition of the original study by Bacon and Egeth (1994), attentional capture was mostly absent (Graves & Egeth, 2015; Kerzel & Barras, 2016; Leber & Egeth, 2006), but some attentional capture may occur during the initial encounters with the distractor (Vatterott & Vecera, 2012; Zehetleitner et al., 2012).

### The original attentional window account

Similar to search mode theory, the attentional window account proposed by Theeuwes (2004) limits attentional capture to certain search tasks. While search mode theory suggests that singleton search results in attentional capture, whereas feature search does not, the original version of the attentional window account claimed that efficient (“parallel”) search results in attentional capture, whereas inefficient (“serial”) search does not. Efficient and inefficient search is associated with search slopes below and above 10 ms/item, respectively (Liesefeld & Muller, 2020; Wolfe & Horowitz, 2004). According to Theeuwes (2004), singleton search is efficient and associated with a large attentional window that encompasses all elements in the display. In contrast, feature search is inefficient and associated with a smaller attentional window. As a result, salient distractors may not be contained inside the attentional window, which eliminates attentional capture. However, feature search was sometimes found to be efficient but attentional capture was absent (Bacon & Egeth, 1994; Kerzel & Barras, 2016; Leber & Egeth, 2006). The latter results are consistent with a bulk of studies on feature search where attentional capture was absent. Importantly target and distractor colors were fixed in these studies (Studies #9–#15 in Table 1; Bacon & Egeth, 1994; Barras & Kerzel, 2016; Graves & Egeth, 2015; Leber & Egeth, 2006; Stilwell & Gaspelin, 2021; Theeuwes, 2004; Wang & Theeuwes, 2020). When target and distractor colors were randomly swapped, attentional capture in feature search was present (Studies #6–#8; Barras & Kerzel, 2016; Gaspelin & Luck, 2018b; Graves & Egeth, 2015), suggesting that participants can only ignore the color distractor when the target has a fixed combination of color and shape.

**Table 1:** Overview of previous studies on attentional capture in feature search

			Unique NTs					
#	Study	Exp.	#	Duplicated	Set Size	Slope	Color	Distractor
1	Gaspelin et al. (2015)	4	3		4		fixed	+
2	Theeuwes (2004)	1	2	no	12, 20	n.s.	fixed	–
3	Wang and Theeuwes (2020)	1	3	yes	6, 10		fixed	–
4	Stilwell and Gaspelin (2021)	3	3	yes	10		fixed	–
5	Zehetleitner et al. (2012)	1	2	no	5 ,9	6	fixed	–
6	Barras and Kerzel (2016)	1	2	no	5, 9	n.s.	random	–
7	Gaspelin and Luck (2018a, 2018b)	1	3		4		random	–
8	Graves and Egeth (2015)	1	4		5		random	–
9	Bacon and Egeth (1994)	3	0, 1, 2	no	5, 7, 9	~6	fixed	n.s.
10	Barras and Kerzel (2016)	1	2	no	5, 9	n.s.	fixed	n.s.
11	Graves and Egeth (2015)	1	4		5		fixed	n.s.
12	Leber and Egeth (2006)	1	2	no	5, 9	3	fixed	n.s.
13	Theeuwes (2004)	2	3	no	5, 9	~12	fixed	n.s.
14	Wang and Theeuwes (2020)	1	3		4		fixed	n.s.
15	Stilwell and Gaspelin (2021)	1-2	4	yes	10, 30		fixed	n.s.

*Note.* In feature search, unique nontargets (NTs) were shown in addition to the target. We indicate the number of unique nontarget shapes and whether these NTs were duplicated at larger set sizes. For experiments with a manipulation of set size, we indicate the search slope if it was significant and indicate “n.s.” otherwise. Target and distractor color were either fixed or swapped randomly. Interference by the color distractor is indicated by – and facilitation by +. Nonsignificant interference is indicated by “n.s.”

### The modified attentional window account

In the current contribution, we deviate from the original version of the attentional window account by suggesting a continuous relation between the size of the attentional window and attentional capture. In contrast, the original version of the attentional window account assumed an almost dichotomous distinction between efficient and inefficient search. However, this dichotomy is inconsistent with the underlying theory. The basic assumption was that a salient distractor can only disrupt visual search inside the attentional window. As a small attentional window is less likely to contain the distractor than a large attentional window, attentional capture is expected to decrease continuously when the attentional window gets smaller and search becomes less efficient. That is, the modified attentional window account predicts less capture in feature than in singleton search whenever search is less efficient.

### The current study

We evaluated search mode theory and the modified version of the attentional window account through manipulations of search efficiency and expectations about the search type. First, we compared singleton and feature search where singleton search was more efficient than feature search. Two different groups of participants performed singleton and feature search, creating strong expectations about the search type. As a result, participants may have used a search strategy based on saliency (“singleton detection mode”) or on target features (“feature search mode”), resulting in more attentional capture in singleton than feature search. While the search mode and attentional window accounts agree that there will be more attentional capture in singleton than feature search, they disagree on the causes. According to the modified attentional window account, attentional capture is proportional to search efficiency. Because search efficiency is higher in singleton than feature search, more attentional capture is expected. With the exception of Theeuwes (2004), however, none of the previous studies have provided an adequate test of the latter hypothesis because search slopes in these studies did not differ between feature and singleton search or were close to zero (Bacon & Egeth, 1994; Kerzel & Barras, 2016; Leber & Egeth, 2006). Second, we manipulated the similarity between target and nontarget shapes. We expect search efficiency to decrease when target and nontarget shapes are similar because the target is less salient. According to search mode theory, the saliency of the target is important in singleton search because participants apply a saliency-based search strategy. Search mode theory therefore predicts more attentional capture when target saliency is low (as with high target–nontarget similarity), which has been confirmed by Barras and Kerzel (2017). For feature search, search mode theory predicts no effect of target–nontarget similarity because search is not based on saliency, but on the exact target features. This prediction will be tested in the current study. In contrast, the modified attentional window account does not consider search type, but only search efficiency. Therefore, it predicts more attentional capture when search efficiency is high (as with low target–nontarget similarity), regardless of search type.

## Experiment 1

**Fig. 1:**
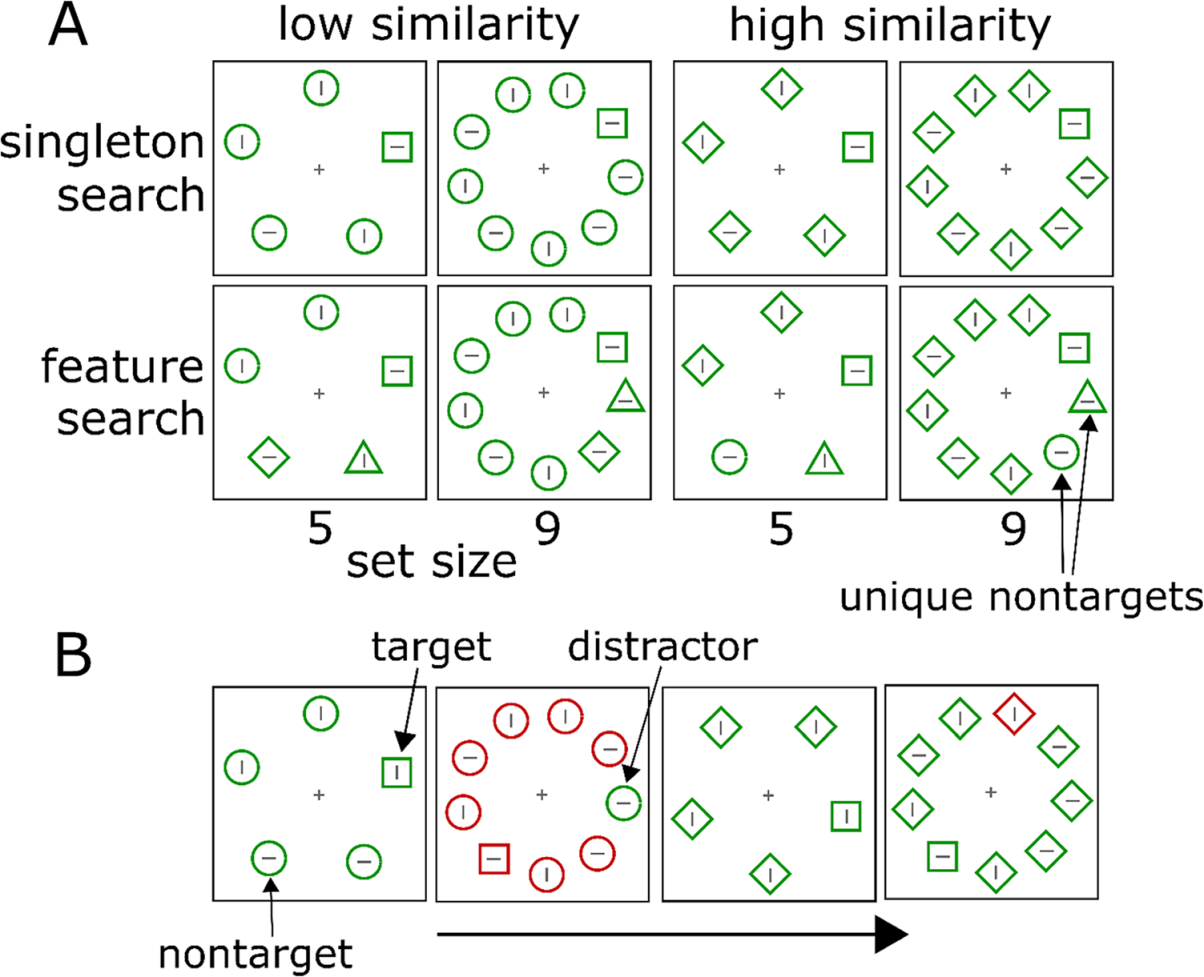
Sample stimuli. Participants searched the square and indicated the orientation of the line inside the square. Panel A illustrates the main experimental manipulations. In Experiment 1, one group of participants performed singleton search and another performed feature search. In Experiment 2, singleton and feature search varied unpredictably from trial to trial. Target–nontarget similarity was manipulated by showing a majority of circle or diamond nontargets. The set size was either 5 or 9. The target square in the illustration is shown approximately on the 2 o’clock position, but positions were random in the experiment. In the sample displays illustrating feature search, the unique nontargets follow the target in clockwise direction (triangle, diamond/circle). Panel B shows a sequence of search displays in the singleton search condition where majority color and target–nontarget similarity changed randomly. (Color figure online)

We manipulated two variables to change search efficiency (see Fig. 1a). First, we asked one group of participants to perform singleton search and another group to perform feature search. According to pilot data, search was less efficient in feature than singleton search, which provides an opportunity to test the modified attentional window account. Second, we manipulated target–nontarget similarity and expected lower search efficiency with similar than dissimilar nontargets. The modified attentional window account predicts less attentional capture with similar nontargets because search efficiency is lower. In contrast, search mode theory predicts more attentional capture with similar nontargets, but only in singleton search. In “singleton detection mode,” participants search for the most salient stimulus and when the saliency of the target is reduced because the nontargets are similar, attentional capture by the color distractor is expected to increase.

In addition, we compared fixed and variable target–nontarget similarity to evaluate whether unpredictable changes of the nontarget shapes would increase attentional capture, similar to random swaps of color. In all conditions, the majority color changed randomly from trial to trial (see Fig. 1b) to induce attentional capture in both singleton and feature search (Gaspelin & Luck, 2018b; Graves & Egeth, 2015; Kerzel & Barras, 2016). With unpredictable target color, participants cannot establish a consistent top-down search goal, which allows for attentional capture in feature search. In all conditions, we manipulated the set size between 5 and 9 items to calculate search slopes. The color distractor was present on 50% of the trials and never coincided with the target shape.

### Methods

#### Participants

First-year psychology students at the University of Geneva participated for class credit. In a pilot study using similar stimuli, we observed that the three-way interaction between search type, target–nontarget similarity, and distractor presence had an effect size of η_p_^2^ = .116 (data available on request). To find an effect of this size in a mixed-factors design with an alpha of .05 and a power of 0.8, a total of 18 participants is necessary according to G*Power 3.1 (Faul et al., 2009). As we were interested in subtle differences between search modes and there were many factors in the experimental design, we settled on a sample size of 48, which would allow for the detection of an effect size of η_p_^2^ = .081. In the final sample, there were 24 students in the group with singleton search (six males, age: *M* = 20.5 years, *SD* = 2.3) and another 24 in the group with feature search (four males, age: *M* = 20.4 years, *SD* = 2.7). To arrive at 48 valid datasets, we had to replace four participants. Three participants had to be replaced because their choice error rate exceeded the limit of 10% indicated in the instructions (remaining sample: *M* = 3.6%, *SD* = 1.8%) and one participant was replaced because many responses were outside the response window of 2,000 ms (7% vs. remaining sample: *M* = 0.6%, *SD* = 0.8%). All students reported normal or corrected-to-normal vision. The study was approved by the ethics committee of the Faculty of Psychology and Educational Sciences and was carried out in accordance with the Code of Ethics of the World Medical Association (Declaration of Helsinki). Informed consent was given before the experiment started.

#### Apparatus

A 22.5-inch VIEWPixx Light monitor (1,920 × 1,200 pixels, 100 Hz, VPixx Technologies Inc., Saint-Bruno, Canada) was used to present the stimuli. Colors and luminance were specified in CIE1931 xyY-coordinates. The xy-coordinates of the stimuli were red = (0.668, 0.312), green = (0.095, 0.737), and gray = (0.276, 0.35). The luminance was Y = 15 cd/m^2^ for all stimuli. The background was black. Color measurements were performed with a ColorCAL MKII colorimeter (Cambridge Research Systems, Rochester, Kent, UK). Viewing distance was maintained by a chin/forehead rest at 66 cm. Responses were collected on a RESPONSEPixx Handheld 5-button response box (VPixx Technologies Inc., Saint-Bruno, Canada), which had four buttons arranged in a diamond shape and one button in the center.

#### Stimuli

Unless otherwise noted, a gray fixation cross (0.4° × 0.4°) was shown in the center of the screen. The search display contained geometric shapes at an eccentricity of 3.5° (center-to-center), drawn in 0.07° wide lines. The perimeter of the angled shapes (square, diamond, triangle) was similar to the circumference of the circle, which had a diameter of 1.5°. The gray lines inside the geometrical shapes were 1.2° long. The geometric shapes were equally spaced on a circular array around fixation, but the rotation of the whole array was random (with a granularity of 1° of rotation). The square was always the target. In singleton search, nontargets were either all circles or all diamonds. Circle nontargets were dissimilar from the square and diamond nontargets were similar to the square, which corresponds to low and high target–nontarget similarity (von Grunau et al., 1994). In feature search, two of the nontargets were unique shapes. With dissimilar nontargets, the majority of nontargets were circles and one triangle and one diamond were added. With similar nontargets, the majority of nontargets were diamonds and one triangle and one circle were added. If present, the distractor was a single nontarget shape in a color different from the others. The majority color was either red or green and varied randomly from trial to trial.

#### Procedure

Trials started with the presentation of the fixation display for a randomly determined duration between 750 and 1,000 ms. Then, the search display was presented until a response was registered. Participants were instructed to look for the target shape without moving their eyes and to indicate the orientation of the line inside the target shape. Horizontal and vertical lines were mapped onto the left and top keys of the RESPONSEPixx response box, respectively. Participants were told that the color of the shapes was irrelevant and should be ignored. They were asked to respond as rapidly as possible while keeping the error rate below 10%. Performance feedback was given after blocks of 64 trials in a self-terminated break of at least 2,000 ms. Visual error feedback was given immediately after choice errors and RTs outside the response window of 2,000 ms.

#### Design

The experimental design was the same in groups performing singleton and feature search. The target shape was always a square and the color distractor never coincided with the target. In singleton search, the nontarget shapes were uniform and target–nontarget similarity was manipulated by changing all nontarget shapes. In feature search, only the majority of nontargets was changed because there were unique nontargets. The order of target–nontarget similarity was manipulated in blocks of 256 trials. Target–nontarget similarity either varied randomly from trial to trial or was fixed. There were two blocks with fixed similarity (low, high) and two blocks with random similarity. Blocks with fixed and random order alternated and the order of blocks was counterbalanced across participants. The 32 combinations of target–nontarget similarity (low, high; applicable only in blocks with random order), distractor presence (present, absent), set size (5, 9), majority color (red, green), and orientation of the target line (horizontal, vertical) were presented once in a mini-block of trials. The order of conditions was random. There were 8 mini-blocks × 32 combinations = 256 trials in each of the four experimental blocks (total of 4 × 256 = 1,024 trials). Sixty-four practice trials were performed before the experiment started.

### Results

The raw data, tables with the mean RTs and the full results of the analysis of variance (ANOVA) are available in the open science framework (https://osf.io/czk9u/). Before calculating individual mean RTs, we removed choice errors (3.6%), responses outside the response interval of 2,000 ms (0.6%) and trials with RTs that were 2.5 standard deviations above the respective condition mean (2.5%). The choice error rate was too low to permit meaningful analyses. In particular, 35% of the cells of the data matrix had values of 0%. Therefore, we only analyzed mean individual RTs and conducted a 2 (search type: singleton, feature) × 2 (target–nontarget similarity: low, high) × 2 (order of target–nontarget similarity: blocked, random) × 2 (set size: 5, 9) × 2 (*n* – 1 majority color: different, same) × 2 (distractor presence: present, absent) mixed ANOVA. Search type was the only between-participant factor.

The six-way mixed-factors ANOVA yielded 15 significant effects, which are summarized in Table 2. None of the remaining effects reached significance (*p*s > .077). Table 2 highlights the interactions of the highest order for each combination of factors. For instance, the four-way interaction of Similarity × Set Size × Order × Distractor Presence (second entry under interactions in Table 2), subsumes the two-way interactions of Similarity × Set Size, And Set Size × Distractor Presence. We will mostly limit our discussion to interactions of the highest order because conclusions from the lower-ranking interactions and main effects cannot be drawn without considering these interactions.

**Table 2:** Results of the analysis of variance in Experiments 1 and 2

	Experiment 1				Experiment 2			
	*F*(1, 46)	*p*	η_p_^2^		*F*(1, 27)	*p*	η_p_^2^	
Main effects:								
Search type	31.664	< .001	.408		142.65	< .001	.841	
Set size	305.22	< .001	.869		80.25	< .001	.748	
Similarity	37.11	< .001	.447		83.20	< .001	.755	
Distractor pres.	131.67	< .001	.741		51.34	< .001	.655	
*n* – 1 majority color	47.42	< .001	.508		34.03	< .001	.558	
Interactions:								
Search Type × Set Size	52.10	< .001	.531		44.18	< .001	.621	Fig. 2
Similarity × Set Size × Order × Distractor Pres.	6.09	.017	.117		-	-	-	Fig. 3
Similarity × Set Size × Distractor Pres.	0.83	.366	.018		11.54	.002	.299	Fig. 3
- Similarity × Set Size	37.21	< .001	.447		37.84	< .001	.584	
- Set Size × Distractor Pres.	14.82	< .001	.244		21.27	< .001	.441	
*n* – 1 Majority Color × Distractor Pres.	49.25	< .001	.517		27.05	< .001	.501	Fig. 4
*n* – 1 Majority Color × Similarity	4.71	.035	.093		6.21	.019	.187	Table 3
Search Type × Similarity × Distractor Pres.	11.90	< .001	.205	≠	1.66	.208	.058	Fig. 5
- Search Type × Distractor Pres.	9.63	.003	.173	≠	0.15	.704	.005	
- Search Type × Similarity	8.28	.006	.152		52.65	< .001	.661	
- Similarity × Distractor Pres.	15.66	< .001	.254		6.15	.020	.184	

*Note.* Higher-order interactions are shown in Figs. 2, 3, 4 and 5 and Table 3. “Similarity” refers to “target–nontarget similarity.” The order of target–nontarget similarity (“order”) was only manipulated in Experiment 1

#### Control analysis

**Fig. 2:**
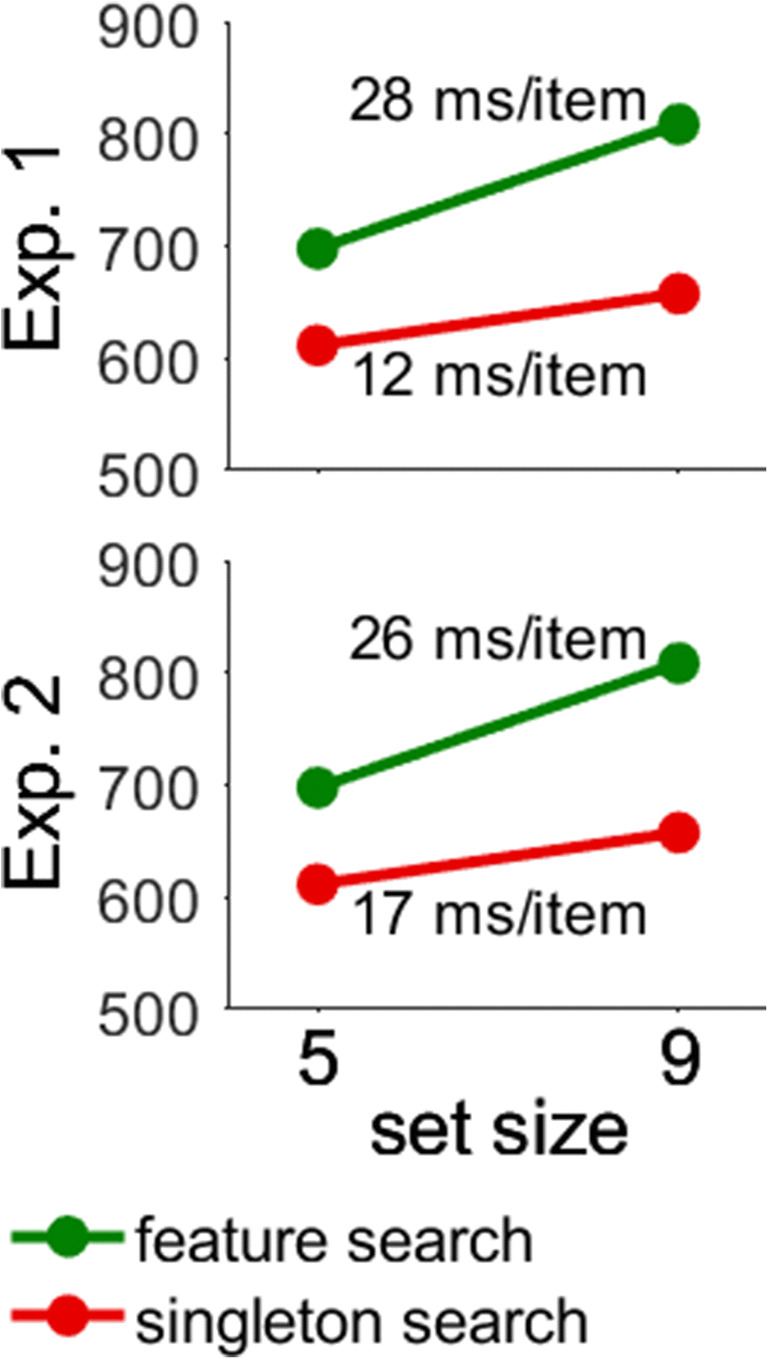
Interaction of search type and set size in Experiments 1 and 2. The search slopes were larger in feature than singleton search. The *y*-axis shows reaction times in milliseconds. Error bars showing the between-participant standard error of the mean were smaller than the symbols. (Color figure online)

Before turning to the main results, we evaluate whether our manipulations of search type and similarity worked as expected. First, we expected search to be more efficient in singleton than in feature search. Figure 2 shows the predicted interaction of Search Type × Set Size. As an index of search efficiency, we calculated the difference in RTs between Set Sizes 5 and 9 and divided the difference by 4. The result is the search slope in ms/item. Search slopes were smaller in singleton than feature search (12 vs. 28 ms/item), as suggested by the modified attentional window account. Both search slopes were significantly different from zero, *t*s(23) > 7.11, *p*s < .001, Cohen’s *d*_*z*_ > 1.34, and above the criterion of 10 ms/item for efficient search (Wolfe & Horowitz, 2004).

**Fig. 3:**
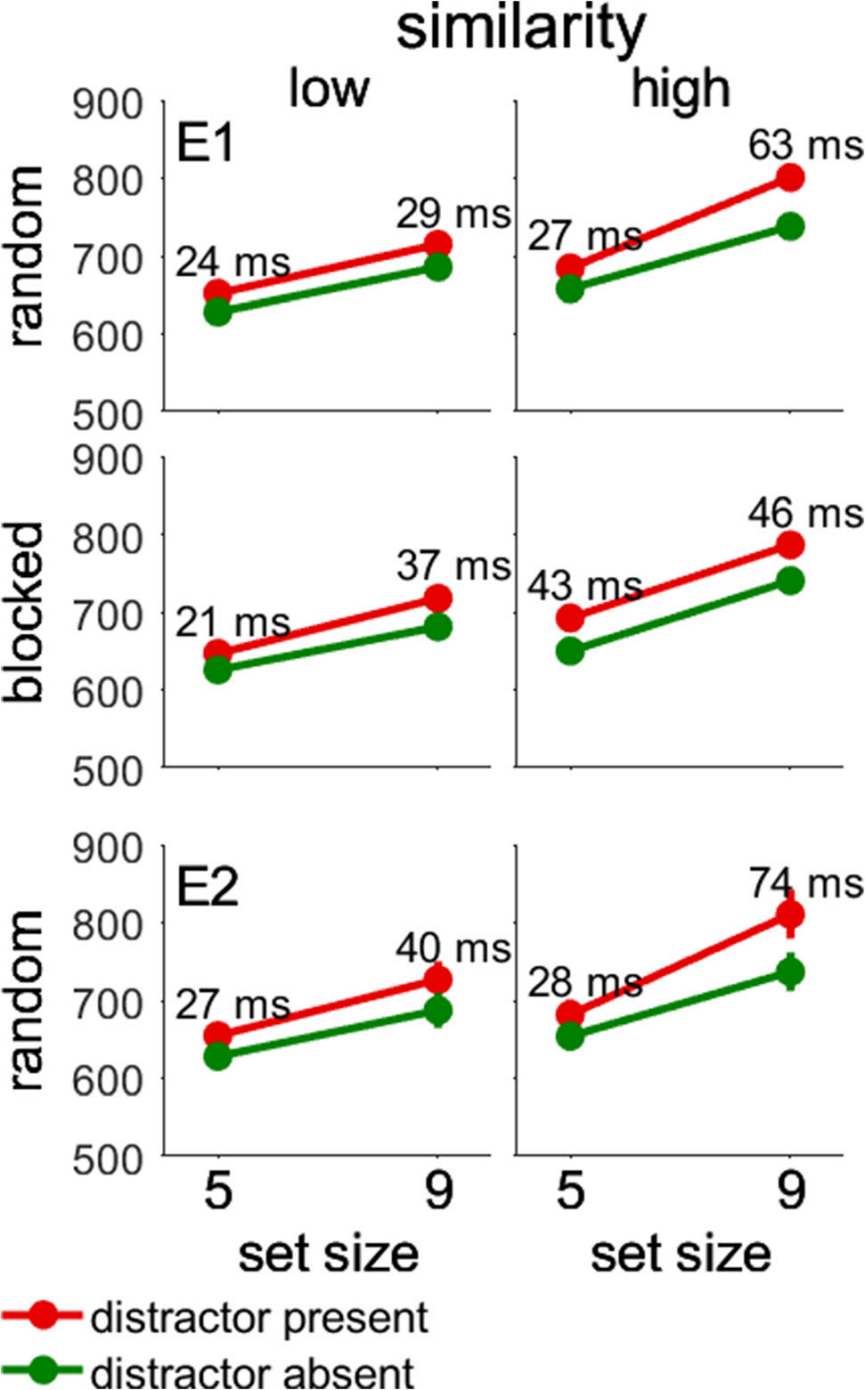
Interaction of set size, similarity, and distractor presence in Experiments 1 and 2. In Experiment 1, the order of target–nontarget similarity (random, blocked) was part of the interaction. In Experiment 2, target–nontarget similarity was always random. The *y*-axis shows reaction times in milliseconds. Error bars showing the between-participant standard error of the mean may be smaller than the symbols. (Color figure online)

Second, we assumed that search would be more efficient with low than high target–nontarget similarity. The interaction of Similarity × Set Size showed that this was indeed the case. The upper four graphs of Fig. 3 show that slopes were smaller with low than high target–nontarget similarity. Collapsed across the other factors, slopes were 15 ms/item with low similarity and 24 ms/item with high similarity, confirming that search became less efficient when the target was similar to the nontargets. However, the interaction of Similarity × Set Size was further modulated in the four-way interaction of Similarity × Set Size × Order of Target–Nontarget Similarity × Distractor Presence, which we had not predicted. The reason for the interaction was that distractor interference was larger in one condition compared with the rest. Notably, distractor interference was larger with random order, high similarity, and set size of 9 compared with the remaining conditions (interference of 63 ms vs. interference <= 46 ms). We do not have an explanation for this pattern of results, but it is unrelated to our experimental hypotheses.

**Fig. 4:**
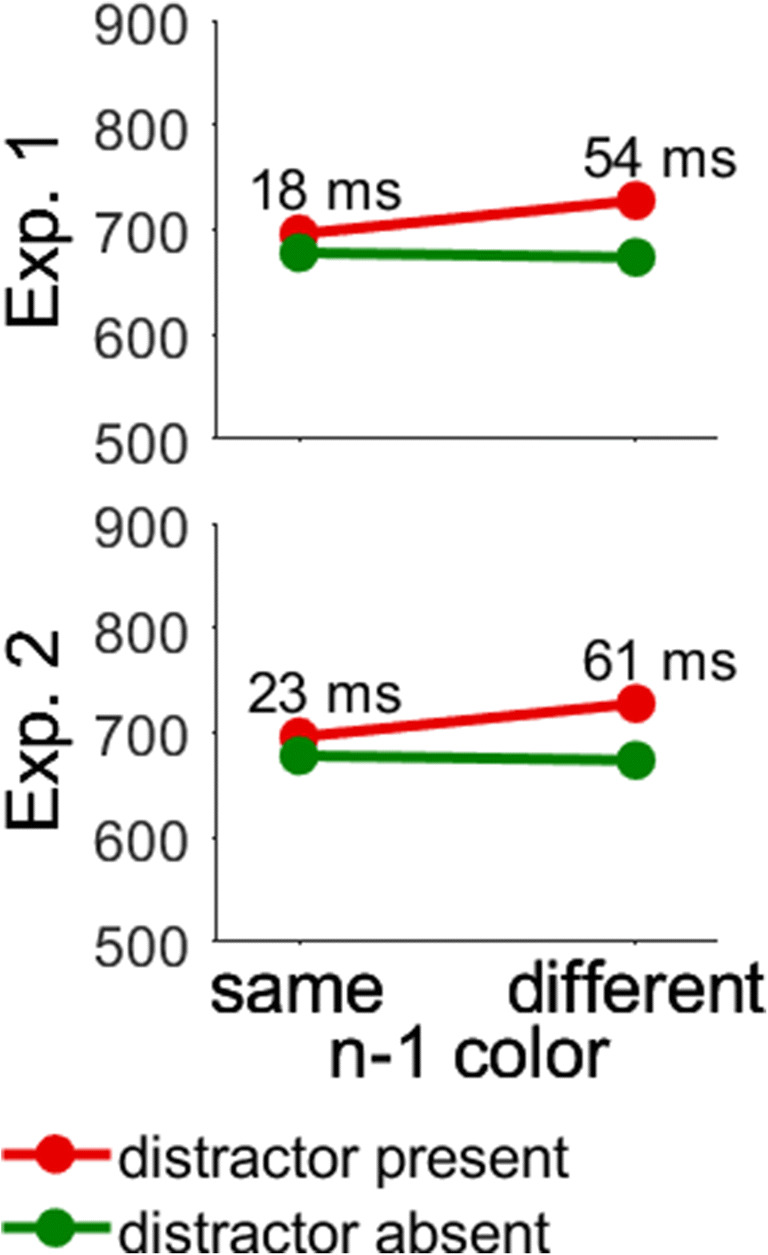
Interaction of majority color in trial *n* – 1 and distractor presence in Experiments 1 and 2. The *y*-axis shows reaction times in milliseconds. Error bars showing the between-participant standard error of the mean were smaller than the symbols. (Color figure online)

Finally, Fig. 4 shows effects of the majority color on the preceding trial (*n* – 1). The interaction of *n* – 1 Majority Color × Distractor Presence replicates previous studies (e.g., Gaspelin & Luck, 2018b; Graves & Egeth, 2015; Hickey et al., 2011; for review, see Ramgir & Lamy, 2021). Distractor interference was larger when the majority color changed than when it was repeated (54 vs. 18 ms). However, distractor interference was significant in both cases. That is, when the majority color on trial n – 1 was different from the majority color on trial *n* (54 ms), *t*(47) = 12.30, *p* < .001, Cohen’s *d*_*z*_ = 1.78, and when it was the same (18 ms), *t*(47) = 4.22, *p* < .001, Cohen’s *d*_*z*_ = 0.61. Further, there was an interaction of *n* – 1 Majority Color × Similarity, which was not predicted. Table 3 shows that RTs generally decreased when the *n* – 1 majority color was the same compared with when it differed, but the decrease was more pronounced on trials with high compared with low target–nontarget similarity.

**Table 3:** Reaction times (ms) as a function of the majority color on trial *n* – 1 (different, same) and target–nontarget similarity (low, high)

		*n* – 1 majority color		
Exp.	similarity	different	same	difference
1	low	674	664	10
	high	728	710	18
2	low	679	669	9
	high	733	708	25

*Note.* The last column shows the difference in reaction times between different and same *n* – 1 majority colors

#### Theoretically relevant analysis

**Fig. 5:**
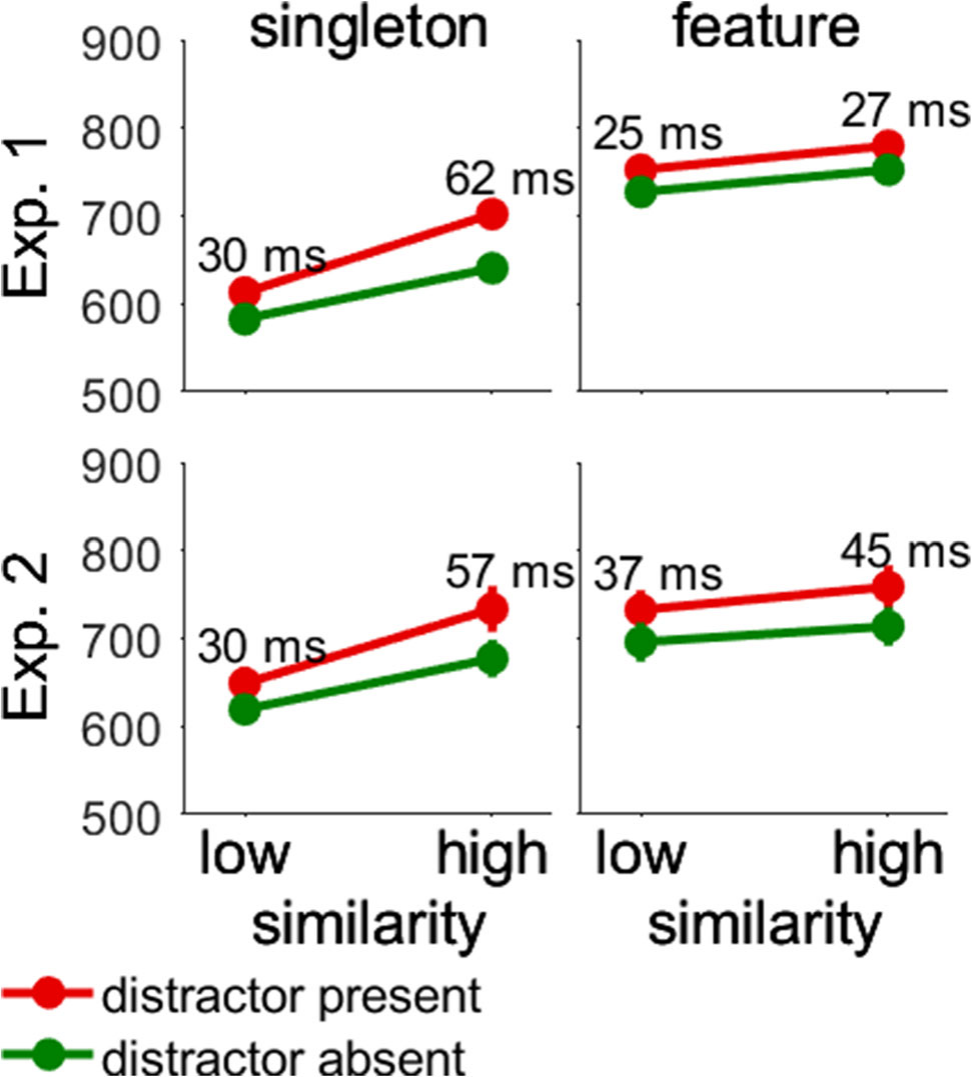
Interaction of search type, similarity, and distractor presence in Experiments 1 and 2. The interaction was significant in Experiment 1, but not in Experiment 2. In Experiment 1, singleton and feature search were performed by separate groups of participants. In Experiment 2, singleton and feature search alternated unpredictably in a single group of participants. The *y*-axis shows reaction times in milliseconds. Error bars showing the between-participant standard error of the mean may be smaller than the symbols. (Color figure online)

The main results of the present experiment are shown in Fig. 5. The interaction of Search Type × Distractor Presence indicated that there was overall more distractor interference in singleton search than in feature search (46 vs. 26 ms), *t*(46) = 3.10, *p* = .003, Cohen’s *d*_*s*_ = 0.90. The larger interference in singleton than feature search is consistent with the modified attentional window account because search was more efficient in singleton search. However, it is also consistent with search mode theory, which claims that search for a singleton target increases distractor interference because participants search for any singleton. The two-way interaction was further qualified by the three-way interaction of Search Type × Similarity × Distractor, which provided a more conclusive test between the two accounts. The interaction indicates that distractor interference was larger with similar compared with dissimilar nontargets in singleton search (62 vs. 30 ms), *t*(23) = 5.68, *p* < .001, Cohen’s *d*_*z*_ = 1.17, but not in feature search (27 vs. 25 ms), *t*(23) = 0.34, *p* = .74, Cohen’s *d*_*z*_ = 0.07. This pattern is consistent with search mode theory and suggests that participants searched for the most salient stimulus in singleton search, which increased attentional capture when the target was more similar to the nontargets and therefore less salient. In contrast, attentional selection was guided by top-down search goals in feature search, which eliminated effects of target–nontarget similarity. In contrast, the modified attentional window account predicted more interference with dissimilar nontargets and high search efficiency regardless of search type, which is inconsistent with the results.

### Discussion

We evaluated predictions of the modified attentional window and search mode accounts. The modified attentional window account predicts reduced attentional capture in conditions where the attentional window is small and search efficiency is low. Consistently, we found less attentional capture in feature than singleton search. However, this result is also consistent with search mode theory, which claims that the search strategy was saliency-based in singleton search, which resulted in more attentional capture than in feature search. Further, the modified attentional window account predicted reduced attentional capture with similar nontargets because search was less efficient. However, effects of target–nontarget similarity were not in the predicted direction and additionally depended on search task. In singleton search, we observed more attentional capture when the target was similar to the nontargets, replicating Barras and Kerzel (2017). In feature search, attentional capture was independent of target–nontarget similarity. While at odds with the modified attentional window account, the results are consistent with search mode theory. According to search mode theory, participants searched for a salient stimulus in singleton search. With similar nontargets, the target became less salient and attentional capture by the more salient color singleton increased. In feature search, however, search mode theory predicts no effect of target–nontarget similarity because saliency plays no role and selection is controlled by top-down search goals. Thus, search mode theory makes correct predictions for effects of target–nontarget similarity, whereas the modified attentional window account does not. However, both accounts can explain the difference between singleton and feature search.

## Experiment 2

In Experiment 1, feature search and singleton search were associated with different search displays. Therefore, it may be that any difference between singleton and feature search resulted from search displays and not from search strategies. To avoid this ambiguity, previous research compared different search strategies with equal search displays. For instance, Bacon and Egeth (1994, Experiment 3) interspersed a minority of singleton search displays in their feature search condition (33% singleton vs. 66% feature search displays) and found that interference from the color distractor was eliminated although the same displays resulted in significant interference in a pure singleton search condition. Another approach is to train participants in feature or singleton search and to test them on the same singleton search displays (Kerzel & Barras, 2016; Leber et al., 2009; Leber & Egeth, 2006; Zehetleitner et al., 2012). In Experiment 2, we opted for yet another solution. We randomly mixed 50% singleton and 50% feature search displays, which prevented participants from applying a consistent search strategy. We therefore expect effects of search type on attentional capture to disappear. According to search mode theory, the interactions of Search Type × Distractor Presence and the interaction of Search Type × Similarity × Distractor Presence should be strongly reduced because search modes can no longer be established. In contrast, the modified attentional window account predicts no changes if differences in search efficiency are preserved.

### Methods

The methods were as in Experiment 1, with the following exceptions. A key finding of Experiment 1 was the interaction of search type, similarity, and distractor presence, which had an effect size of η_p_^2^ = .205. To find this interaction in a within-subject design with power = .8 and alpha = .05, only 10 participants are necessary. We opted for a sample size of 28 (four males, age: *M* = 21.8 years, *SD* = 6.1), which would allow us to detect effect sizes as small as η_p_^2^ = .07. In addition, this sample size is similar to the 24 participants per group in Experiment 1. The displays were as in Experiment 1, but singleton and feature search alternated randomly. Participants worked through 1,024 trials, divided into four blocks of 256 trials. None of the participants had participated in Experiment 1. One participant was replaced because of an error rate larger than 10% (remaining sample: *M* = 4.6%, *SD* = 2.1%).

### Results

Before calculating individual mean RTs, we removed choice errors (4.6%), responses outside the response interval of 2,000 ms (1.2%) and trials with RTs that were 2.5 standard deviations above the respective condition mean (2.5%). We conducted a 2 (search type: singleton, feature) × 2 (target–nontarget similarity: low, high) × 2 (set size: 5, 9) × 2 (n – 1 majority color: different, same) × 2 (distractor presence: present, absent) repeated-measures ANOVA. In contrast to Experiment 1, target–nontarget similarity was always random and search type was a within-participant factor.

The five-way repeated-measures ANOVA yielded 13 significant effects, which are summarized in Table 2. None of the remaining effects reached significance (*p*s > .137). As shown in Table 2, the results were highly consistent between Experiments 1 and 2, with some notable exceptions (highlighted by the unequal sign ≠).

#### Control analysis

Before turning to these exceptions, we will evaluate whether we replicated the main findings from Experiment 1. First, we expected search to be more efficient in singleton than feature search. Figure 2 shows the predicted interaction of Search Type × Set Size. Search slopes were smaller in singleton than feature search (17 vs. 26 ms/item), but significantly different from zero in both cases, *t*s(27) > 7.10, *p*s < .001, Cohen’s *d*_*z*_ > 1.3.

Second, the interaction of Similarity × Set Size showed that search was more efficient when the target was shown with dissimilar nontargets, as in Experiment 1. The two lower graphs of Fig. 3 show that slopes were shallower with low than high target–nontarget similarity. Collapsed across the remaining factors, slopes were 16 ms/item with low similarity and 26 ms/item with high similarity, confirming that search was more efficient when the target was dissimilar from the nontargets. The interaction of set size and similarity was further modulated in the three-way interaction of Similarity × Set Size × Distractor Presence. As in Experiment 1, distractor interference was larger with high similarity and set size of 9 compared with the remaining conditions (interference of 74 ms vs. interference <= 40 ms), but we do not have an explanation for this effect.

Finally, Fig. 4 shows that we replicated effects of intertrial changes of color. The interaction of *n* – 1 Majority Color × Distractor Presence shows that distractor interference was larger when the majority color changed between the previous and the current trial than when it remained the same (61 vs. 23 ms). However, distractor interference was significant with changes of the majority color (61 ms), *t*(27) = 7.48, *p* < .001, Cohen’s *d*_*z*_ = 1.41, and with repetitions (23 ms), *t*(27) = 4.30, *p* < .001, Cohen’s *d*_*z*_ = 0.81. Further, we replicated the interaction of *n* – 1 Majority Color × Similarity, as shown in Table 3.

#### Theoretically relevant analysis

The main difference between Experiments 1 and 2 is shown in Fig. 5. First, we examined the interaction of Search Type × Distractor Presence. In Experiment 1, this interaction showed that there was more interference in singleton than in feature search (46 vs. 26 ms). In Experiment 2, however, interference was about the same in singleton and feature search (43 vs. 41 ms), *t*(27) = 0.38, *p* = .704, Cohen’s *d*_*z*_ = 0.07. Table 2 shows that the effect size of the interaction of Search Type × Distractor Presence decreased from η_p_^2^ = .173 in Experiment 1 to η_p_^2^ = .005 in Experiment 2. One may argue that the interaction was less reliable because fewer trials were available per search mode in Experiment 2 than Experiment 1 (512 vs. 1,024 trials). To rule out this possibility, we reanalyzed only the condition with random similarity in Experiment 1, which balanced the number of trials per search mode in Experiments 1 and 2. However, the interaction remained significant in Experiment 1, *F*(1, 46) = 6.05, *p* = .018, η_p_^2^ = .116, and was larger than in Experiment 2 (η_p_^2^ = .005). This result suggests that search strategies explain the larger interference in singleton than feature search in Experiment 1. When search strategies were unavailable in Experiment 2 because singleton and feature search occurred randomly, attentional capture was no longer stronger in singleton than in feature search. In contrast, search remained more efficient in singleton than feature search, but this difference was not sufficient to induce larger interference in singleton than feature search.

Further, we examined the three-way interaction of Search Type × Similarity × Distractor Presence. In Experiment 1, this interaction was significant which indicated that distractor interference was 32 ms larger with similar than dissimilar nontargets in singleton search, whereas distractor interference was about the same in feature search. This result favored search mode theory because singleton search is based on saliency, whereas feature search is based on top-down search goals. In contrast, the modified attentional window account predicts more interference with dissimilar nontargets regardless of search type, which is inconsistent with the results. Table 2 shows that this interaction was not significant in Experiment 2, *F*(1, 27) = 1.66, *p* = .208, η_p_^2^ = .058, although the study was sufficiently powered based on the results of Experiment 1. Nonetheless, follow-up *t* tests showed that the pattern of results was similar. That is, distractor interference was 27 ms larger with similar than dissimilar nontargets in singleton search (57 vs. 30 ms), *t*(27) = 4.66, *p* < .001, Cohen’s *d*_*z*_ = 0.88, but only 8 ms in feature search (45 vs. 37 ms), *t*(27) = 0.65, *p* = .52, Cohen’s *d*_*z*_ = 0.12. Thus, the pattern of results was similar in Experiments 1 and 2, but the size of the effect was drastically reduced from η_p_^2^ = .205 to η_p_^2^ = .058. To rule out that the smaller effect size resulted from the lower number of trials per condition in Experiment 2, we again restricted the analysis of Experiment 1 to the condition with random similarity. The interaction of Search Type × Similarity × Distractor Presence remained significant in Experiment 1, *F*(1, 46) = 9.03, *p* = .004, η_p_^2^ = .164, and the effect size was still larger than in Experiment 2 (η_p_^2^ = .058). Thus, the modulation of distractor interference by similarity (i.e., the interaction of Search Type × Similarity × Distractor Presence) depended on search strategies, which were unavailable in Experiment 2 because singleton and feature search occurred randomly.

### Discussion

To assess the contributions of search modes, we randomly mixed 50% singleton and 50% feature search. We suggested that this manipulation would destroy expectations about the search type. As a result, participants could no longer apply a consistent search strategy, and search mode theory predicts differences in attentional capture to disappear. We largely confirmed this prediction. In Experiment 1, we found that attentional capture was larger in singleton than feature search, whereas in Experiment 2, attentional capture was comparable between singleton and feature search. Similarly, the interaction with target–nontarget similarity was reliable in Experiment 1, but not in Experiment 2. While the differences between Experiments 1 and 2 are predicted by search mode theory, they are inconsistent with the modified attentional window account. The reason is that search efficiency was comparable between Experiments 1 and 2. Critically, efficiency was higher in singleton than feature search in both experiments (see Fig. 2), but attentional capture was larger in singleton search only in Experiment 1 (see Fig. 5). In addition, search was more efficient when the target was dissimilar from the nontargets in both experiments (see Fig. 3), but the interaction with search type and distractor presence was significant only in Experiment 1 (see Fig. 5). Thus, the pattern of search efficiency was similar in Experiments 1 and 2, but the pattern of attentional capture changed, which is incompatible with the idea that search efficiency alone determines attentional capture.

While the present experiment provides support for search mode theory, our conclusions are limited by the experimental design. Search mode was manipulated between-participant in Experiment 1, but within-participant in Experiment 2. While this design isolates effects of expectations, it prevents direct statistical comparisons. Therefore, we relied on comparisons of effect sizes and statistical significance between experiments, which does not provide conclusive evidence. However, a similar logic was applied by Theeuwes (2004) and Bacon and Egeth (1994). Further, the options for the current research question are limited by the fact that participants continue to use a trained search mode (Kerzel & Barras, 2016; Leber et al., 2009; Leber & Egeth, 2006; Zehetleitner et al., 2012). Therefore, it may be difficult to destroy expectations about the search type once a search mode has been acquired, which makes a within-participant manipulation of expectations difficult.

## General discussion

In two experiments, we assessed predictions of the modified attentional window account and search mode theory. The modified attentional window account holds that attentional capture increases continuously with search efficiency. In Experiment 1, singleton search was more efficient than feature search, and consistently, attentional capture was larger in singleton than feature search. However, this result can also be explained by search mode theory. Search mode theory claims that participants used a saliency-based search strategy in singleton search, which increased attentional capture relative to feature search. To decide between the two accounts, we destroyed expectations about the search type in Experiment 2 by randomly mixing 50% singleton and 50% feature search displays. If search displays cannot be anticipated, participants are unable to apply a consistent search strategy, and search mode theory therefore predicts differences in attentional capture between search displays to disappear. In contrast, the modified attentional window account does not consider search strategies and predicts unchanged results as long as search efficiency remains the same. The results favor search mode theory because differences in attentional capture between singleton and feature search were strongly reduced when search types were randomly mixed. In contrast, the modified attentional window account would still predict less capture in feature than in singleton search because search efficiency continued to be lower in feature search. Further, with singleton search in Experiment 1, we found stronger attentional capture when the target was similar to the nontargets, which is opposite to predictions of the modified attentional window account because search was less efficient. Again, the respective interaction was strongly reduced with unpredictable search types in Experiment 2, which confirms search mode theory but contradicts the modified attentional window account because search efficiency was unchanged. Overall, our results favor search mode theory and are inconsistent with the modified attentional window account.

### Effects of set size on saliency

It is a common assumption that the saliency of a singleton increases with set size because the density of the display is greater, and the computation of local contrast is facilitated. However, analysis of search displays through computational models of saliency demonstrated that singletons are salient even in small search displays (Chang et al., 2021). Nonetheless, there is evidence that search is facilitated with larger set sizes (Bacon & Egeth, 1991; Bravo & Nakayama, 1992; Buetti et al., 2016; Rangelov et al., 2017; Theeuwes, 2004). If the saliency of the target singleton increases at larger set sizes, search efficiency is expected to increase, and the modified attentional window account predicts more attentional capture. In particular, Wang and Theeuwes (2020) found attentional capture in feature search with set sizes of 6 and 10, but not with a small set size of 4 (see Studies #3 and #14 in Table 1). Possibly, the saliency of the target was higher with set sizes of 6 and 10, which allowed for efficient search and resulted in attentional capture. However, set size in Wang and Theeuwes was manipulated across groups of observers and search slopes could not be computed. Therefore, we do not know whether search was more efficient at larger set sizes. Additional evidence against the connection between set size, target saliency, and search efficiency was provided by Stilwell and Gaspelin (2021). With new displays and set sizes of 10 and 30, Stilwell and Gaspelin did not observe attentional capture in feature search, but attentional capture reemerged with the original stimuli from Wang and Theeuwes (see Studies #4 and #15). Future research therefore needs to clarify why certain dense displays result in attentional capture in feature search, whereas others do not.

While feature search with large set sizes sometimes resulted in attentional capture, the opposite was observed for small set sizes, which is difficult to explain if only target saliency is considered. That is, with fixed colors and small set sizes, some studies found RTs on distractor-absent trials to be shorter than RTs on distractor-present trials (see Study #1 in Table 1; Gaspelin et al., 2015; Gaspelin & Luck, 2018a; Kerzel & Burra, 2020; Lamy, Bar-Anan, et al., 2006a). These negative distractor effects were interpreted as resulting from the suppression of salient distractors in conditions where top-down control is strong. However, the random color swaps in the current study prevented strong top-down control as it was impossible to establish a single target representation. An enhanced attentional template may be necessary to resist interference (see Huynh Cong & Kerzel, 2020, 2021). Thus, we neither predicted nor observed facilitation by the distractor in the current study.

Overall, it is unclear whether manipulations of set size truly change target saliency, and it is therefore unclear whether increased capture with large set sizes can be explained by higher search efficiency. In particular, different set sizes are necessary to measure search efficiency, and it is therefore difficult to conclude that search was more efficient at one of these set sizes. To avoid this problem, we manipulated target saliency by changing target–nontarget similarity. As reported in the results, this manipulation had the expected effect as search efficiency dropped when the target was more similar to the nontargets. However, there is a possible caveat. In feature search, the choice of nontargets not only affected the saliency of the target, but also the saliency of the unique nontargets. Thus, it may be that target–nontarget similarity had different effects on singleton and feature search. However, the relevant interaction of Search Type × Similarity × Set Size was not significant, Experiment 1: *F*(1, 46) = 0.54, *p* = .464, η_p_^2^ = .012; Experiment 2: *F*(1, 46) = 1.70, *p* = .203, η_p_^2^ = .059. Thus, the decrease of search efficiency when the target was similar to the nontargets was comparable in singleton and feature search, which strengthens our conclusion that search modes were responsible for the differential effects of similarity in singleton and feature search in Experiment 1.

### Effects of set size on distractor saliency

Similar to effects of set size on target saliency, one may wonder whether the saliency of the distractor increased when the set size was large and the display was dense. If so, search mode theory would predict more attentional capture in singleton search because search is based on saliency whereas no difference is expected in feature search. However, we did not find an interaction of Search Mode × Set Size × Distractor Presence to confirm this prediction (for similar results, see Lamy, Bar-Anan, et al., 2006a; Lamy, Carmel, et al., 2006b). Further, there are reasons to doubt the assumption that the saliency of the color distractor was higher at larger set sizes. In fact, color distractors at small set sizes are already highly salient (Chang et al., 2021). In addition, previous work showed that shorter RTs with color singletons in large set sizes only occurred in search tasks that implied the shape of the color singleton (Bravo & Nakayama, 1992; Buetti et al., 2016; Rangelov et al., 2017). However, the color distractor in our experiments was not associated to any task. Therefore, there is little evidence to suggest that the saliency of the color distractor was higher with larger set size. Possibly, its saliency was already at ceiling with only four nontargets. Further, we found that the increased attentional capture with large set sizes was not universal but depended on target–nontarget similarity and the order of target–nontarget similarity (see Fig. 3). As already stated before, we do not have an explanation for this interaction, which keeps us from drawing firm conclusions.

### Intertrial effects

We observed more interference from the color distractor after changes than repetitions of the majority color, which is consistent with previous research (e.g., Gaspelin & Luck, 2018b; Graves & Egeth, 2015; Hickey et al., 2011; for review, see Ramgir & Lamy, 2021). In a similar vein, previous studies on singleton search have investigated whether changes or repetitions of the target shape affect interference. It was demonstrated that interference from a color distractor was much larger when target and nontarget shapes swapped randomly compared with when they were fixed (Burra & Kerzel, 2013; Lamy, Bar-Anan, et al., 2006a; Lamy, Carmel, et al., 2006b; Lamy & Yashar, 2008; Pinto et al., 2005; Theeuwes, 1991, 1992). Thus, any manipulation that weakens the attentional template for the target appears to increase attentional capture.

Further, we found larger attentional capture after changes of the majority color, but this effect was not modulated by search mode, *p*s > .29. Thus, the larger attentional capture in singleton than feature search in Experiment 1 cannot be chalked up to intertrial effects. Also, intertrial priming cannot explain the occurrence of attentional capture in feature search because attentional capture was also significant after repetitions of the majority color. However, when colors were fixed (and therefore repeated on every trial), many studies found no significant attentional capture in feature search (see Table 1). Again, we attribute significant attentional capture in feature search with random color changes to the incomplete attentional template for the target, which prevented strong top-down guidance and allowed for distractor interference. Further, it made no difference in the current study whether target–nontarget similarity was blocked or changed randomly. That is, it did not matter whether the nontargets were the same from trial to trial or whether they changed randomly. While the absence of an effect must be interpreted with caution, this result suggests that only the trial history of selected stimuli is relevant, whereas the relation between target and nontarget stimuli has no effect. This result is surprising given the sensitivity of attention and working memory to relational information (e.g., Martin & Becker, 2021; Schönhammer et al., 2016).

### Conclusion

We investigated effects of search efficiency to test the attentional window account (Theeuwes, 2004) against search mode theory (Bacon & Egeth, 1994). According to the modified version of the attentional window account, attentional capture increases when search is efficient because the attentional window is large. In our experiments, search efficiency was always higher in singleton than feature search, but contrary to predictions of the modified attentional window account, attentional capture was not always larger in singleton than feature search. In particular, we only found more capture in singleton than feature search when participants performed either singleton or feature search throughout the experiment, but not when singleton and feature search varied unpredictably. Further, effects of target–nontarget similarity contradict the modified attentional window account. Search efficiency decreased when targets were similar to the nontargets. According to the modified attentional window account, low search efficiency should lead to less attentional capture, but we found the opposite in singleton search and no difference in feature search. While inconsistent with the modified attentional window account, the results support search mode theory. Search mode theory claims that participants looked for the most salient stimulus in singleton search, but for a specific stimulus in feature search. When expectations about the search type were destroyed, search mode theory predicts differences in attentional capture to disappear, which was confirmed by our results. Also, search mode theory predicts more attentional capture in singleton search when the target is similar to the nontargets, which we confirmed when search modes could be established. Overall, our results support search mode theory and cast doubts on the validity of the attentional window account.
